# Study protocol for a multi-center RCT testing a group-based parenting intervention tailored to mothers with borderline personality disorder against a waiting control group (ProChild*-SP1)

**DOI:** 10.1186/s13063-022-06531-2

**Published:** 2022-07-23

**Authors:** Charlotte Rosenbach, Nina Heinrichs, Robert Kumsta, Silvia Schneider, Babette Renneberg

**Affiliations:** 1 Department of Education and Psychology, Freie Universität Berlin, Berlin, Germany; 2 Department of Psychology, Universität Bremen, Bremen, Germany; 3 Faculty of Psychology, Ruhr-University Bochum, Bochum, Germany

**Keywords:** Borderline personality disorder, Parenting, Group intervention, Randomized-controlled trial, Study protocol

## Abstract

**Background/aims:**

Borderline personality disorder (BPD) is a severe mental disorder characterized by an unstable sense of self, intense and rapidly changing affect, as well as impulsive and self-destructive behaviors. Interpersonal relationships of individuals with BPD are characterized by marked instability, a lack of dependability, and quick changes between love and hate. For children of individuals with BPD, this can lead to permanent stress and attachment insecurity and an increased risk of adverse physical and mental health development. To reduce dysfunctional parenting and improve positive parenting, and in turn, to promote healthy child development, a group intervention for mothers with BPD was developed. This study aims to evaluate this first disorder-specific parenting intervention for BPD in a randomized controlled trial.

**Method:**

In a parallel-group, two-arm, randomized controlled trial, an initial *N* = 178 mothers diagnosed with BPD and their children aged 6 months to 6 years are assigned to either the parenting intervention or a waiting control group. If taking place, participants of both groups continue their regular treatment for BPD diagnosis (e.g., individual therapy, medication). The primary outcomes are changes in parenting from baseline (day 0) to post intervention (week 12) and follow-up (6 months after group intervention; month 9). The waiting control group can attend the group intervention at the end of all assessments. Participants allocated to the intervention group are expected to show improvement in their parenting and a reduction in child abuse potential. Maternal emotion regulation and mental distress are analyzed as secondary outcomes.

**Discussion:**

Mothers with BPD may need tailored help when reporting difficulties raising their children. The first disorder-specific parenting intervention has been developed to close this gap. ProChild is part of a large government-supported consortium, which aims to investigate different aspects of abuse and maltreatment in childhood and adolescence.

**Trial registration:**

ClinicalTrials.gov NCT04169048. Registered on Nov 19, 2019.

## Introduction

### Background

Borderline personality disorder (BPD) is found in approx. 1.7% of the general population, and 15–28% of patients in inpatient settings [1]. BPD is characterized by a pervasive instability in mood, self-image, and identity. In stressful situations, individuals with BPD frequently react with impulsive or self-harming behavior, easily lose their temper, or engage in self-damaging behavior (e.g., drinking, drug use, self-injury). Additionally, patients with BPD have difficulties engaging in and especially maintaining stable relationships, are hypersensitive to rejection, and often mistrust others. Even though evidence-based treatments for BPD are available [1, 2], and most of the patients recover over the life span [3], especially the years between ages 20 and 35 can be extremely challenging for individuals with BPD. Additionally, a severe and persistent impairment in social functioning has been observed [3]. Nevertheless, for this age typical normative developmental steps, such as getting married and starting a family, are common in BPD, too. This can lead to more stability and a sense of identity on the one hand—but on the other hand to even bigger challenges in managing every day’s life.

The borderline typical “stable instability” of interpersonal problems and emotion regulation [4] affects all daily interactions, including the parents’ relationship with their infant(s). Especially the first years of parenting are often a big challenge for parents with BPD. Factors such as sleep deprivation, changes in daily routines, and setting new priorities put the mothers under great pressure, reinforcing the borderline symptoms (e.g., impulsivity, self-harming behavior).

Considering that a healthy child development depends on the stability and dependability of the caregiver, it is crucial to offer mothers with BPD support in raising their children early on.

The primary goal of the presented randomized controlled trial (RCT) is to test the efficacy of a newly developed parenting group program for mothers with BPD ([5], English Version: [6]). The intervention is designed to promote positive parenting by teaching about the child’s primary needs, coping with parenting stress, emotion regulation, dealing with conflicts, and self-care. As prior research has focused on mothers with BPD and mothers mostly still are the caretakers early in life, the manual is conceptualized for mothers with BPD.

#### Mothers with BPD

An increasing number of studies point to the numerous difficulties mothers with BPD face (for an overview: [7–10]). Mothers with BPD often have difficulties in interpreting their offspring’s mental [11, 12], as well as emotional states [13], and thus have great difficulty responding sensitively to their infant’s needs [14]. A study investigating mother-infant dyads showed that mothers with high BPD traits perceived more negative emotions in their infants than could be objectified [15]. Furthermore, they reacted with more punitive and trivializing (invalidating) parenting behaviors than mothers with low BPD traits [15]. This behavior was mainly predicted by maternal emotion regulation difficulties [15]. In the communication with their children, mothers with BPD show increased hostility [16], more affective dysregulation, as well as more critical and intrusive behaviors, role confusion, and frightened/frightening behaviors than a non-clinical control group [17, 18].

Compared to non-clinical control groups, mothers with BPD feel less competent about parenting and perceive parenting as more stressful and less rewarding [14, 19, 20]. They rate their impact on their infants’ emotions and behavior weaker than a healthy control group does [13]. Additionally, they showed significantly more insecure, preoccupied and unresolved attachment patterns than a normative comparison group [21].

Parental BPD is also associated with a greater risk of child abuse [22, 23], whereas the effects of BPD on abuse potential are mediated by the severity of emotion regulation difficulties [23]. Abuse potential, in turn, is positively associated with child psychopathology [23]. Additionally, families with mothers diagnosed with BPD are often characterized by instability, low family cohesion, as well as frequent drug and alcohol abuse [24]. Further stressors like being a single parent, having low social support in the family, frequently changing partners and unstable households put mothers with BPD under additional personal stress, which may spill over into the parent–child dyad.

#### Children of mothers with BPD

Compared to children of healthy mothers, children of mothers with BPD have a higher risk for emotional and behavioral problems [7, 25]. This is also the case when compared to children of mothers with depressive disorders or cluster C personality disorders [26].

Already at an early stage, the offspring of mothers with BPD show signs of emotion dysregulation. Before the age of one, the infants were less responsive to their mothers [27] and expressed less positive phonation and more negative affect than infants of mothers with no psychopathology [28]. Furthermore, infants of mothers with BPD showed either too high or too low levels of fear expression [29].

Preschoolers and schoolchildren of mothers with BPD show more fear of abandonment [21] and parent–child role reversal [18]. They endure more maltreatment in an interaction task [30] compared to children of a healthy control group. In another study, mothers with BPD reported that their 4–7-year-old children had higher negative affectivity and lower effortful control compared to children of healthy mothers [31]. This was supported by teacher ratings of child behavior and child self-report (story completion task).

In adolescence, disruptive behavior, attention deficit/hyperactivity disorder (ADHD) and borderline symptoms appear to be more prevalent in children of mothers with BPD than in children of healthy mothers [32, 33]. Maternal borderline symptoms are associated with insecure, fearful attachment and poor self-perception in their youth offspring [34]. Mahan and colleagues [35] pointed at the link between maternal affective instability and adolescent’s affective instability, emphasizing the assumption of intergenerational transmission of BPD traits.

In sum, from infancy up to adolescence, children of mothers with BPD have a higher risk for emotional and behavioral problems than children of healthy mothers or children of mothers with other mental disorders such as depression.

Whereas the parental influence is strong in early childhood, peers and the social context gain relevance with age. Nevertheless, these early years are crucial for formative experiences and shape long-lasting ideations and schemes regarding one’s own needs, identity, and the formation of interpersonal relationships. To promote a healthy child development, the group intervention “Parenting Skills for Mothers with Borderline Personality Disorder” (5, English Version: 6) addresses mothers of young children between 6 months and 6 years. Consequently, dysfunctional parenting can be reduced or even prevented as early as possible.

### Objectives

The study *Parenting Skills for Mothers with BPD* is part of the consortium ProChild (“Preventing maltreatment and *pro*moting mental health in *child*ren of mothers with borderline personality disorder”), funded by the Federal Ministry of Education and Research (Bundesministerium für Bildung und Forschung; BMBF; 001KRI805A). ProChild aims across five subprojects to:

1. Evaluate the group intervention described below in a randomized controlled trial (subproject 1, clinical registration: NCT04169048),
2. Identify the specific characteristics and interactional patterns of children and their mothers with BPD compared to dyads with maternal anxiety or depressive disorders and dyads with healthy mothers (subprojects 2 and 3, non-intervention study registration: DRKS00020460), and
3. Assess and derive recommendations for improved cooperation between childcare institutions, parents, and practitioners (subproject 5).

Subproject 4 analyzes epigenetic mechanisms in mothers and their children over time.

The current study protocol relates to the clinical trial (subproject 1). We will conduct and evaluate the first disorder-specific parenting intervention for mothers with BPD in a multi-center randomized controlled trial. The intervention is conceptualized as an add-on intervention to a completed or ongoing individual therapy focusing on the borderline symptomatology of the mother. Participation in the group program does not substitute individual psychotherapy. All participants must either have already completed or actually be in an individual treatment for their BPD.

The primary hypothesis is that the intervention has positive effects on parenting, and decreases child abuse potential directly after the intervention and at 6-month follow-up compared to a waiting control group. Secondary, it is hypothesized that the intervention reduces parental stress and enhances perceived parental competences. As well, an improvement in maternal emotion regulation and a reduction of mental distress is expected.

### Trial design

The trial is designed as a randomized, controlled, multi-center trial with two parallel groups. Randomization will be performed as clustered randomization with a 1:1 allocation by RedCap software for each study center separately.

## Methods: participants, interventions, and outcomes

### Study design, recruitment, and eligibility

Participants are recruited via three academic study centers in the German cities of Berlin, Bochum, and Bremen. Each study center cooperates with different in- and outpatient facilities, assisted living facilities, and individual therapists, through which participants with BPD are recruited. Most of these services are covered by the public health insurance, so that we expect the sample to be representative for mothers with BPD living in Germany. All participants will first be screened by a short telephone interview regarding their study eligibility (inclusion and exclusion criteria see below). When meeting the inclusion criteria, participants are invited to a face-to-face diagnostic interview (Structured Clinical Interview for DSM 5; SCID CV & SCID PD; German version: [36]; modules: mood disorders, psychotic symptoms, substance use disorders, anxiety disorders, posttraumatic stress disorder, BPD) to assess BPD symptoms and comorbid diagnosis as well as child-related psychopathology (subproject 2). Staff members who received a 2-day training and are supervised regularly conduct all diagnostic interviews. When still meeting inclusion criteria, participants obtain a personal link to complete several web-based questionnaires. Afterwards, they are invited to a first assessment with their child, where variables of subprojects 2, 3, and 4 are assessed. Participants are then randomized to the intervention or the waiting control group. All questionnaires and assessments are repeated post-interventional (week 12) and at follow-up 6 months later (month 9). After completing all assessment points, mothers randomized to the waiting control group are offered to participate in the parenting program (see also Table 1 SPIRIT schedule of enrollment, interventions, and assessments/participant timeline).

**Table 1:** SPIRIT schedule of enrollment, interventions, and assessments

Timepoint	Study period																
	Enrolment		Allocation	Post-allocation													
				Intervention period													
			Basement (day 0)	Week 1	Week 2	Week 3	Week 4	Week 5	Week 6	Week 7	Week 8	Week 9	Week 10	Week 11	Week 12	Post-assessment (week 12)	Follow-up assessment (month 9)
Enrolment																	
Eligibility screen	x																
Informed consent	x																
Randomization BPD		x															
Intervention				x	x	x	x	x	x	x	x	x	x	x	x		
Assessments																	
Socio-demographics		x															
Diagnosis		x															
Primary outcomes			x													x	x
Secondary outcomes			x													x	x
Additional measures			x						x							x	x
Child protection routines		x		x	x	x	x	x	x	x	x	x	x	x	x		
Intervention related measures				x	x	x	x	x	x	x	x	x	x	x	x		

Participants must provide written, informed consent before participating in the face-to-face diagnostic interview. All assessments are rewarded monetary and children receive a small gift.

Inclusion criteria:

- Mothers with diagnosed BPD having children aged 6 months to 6 years (according to the age range addressed in the training “Parenting skills for mothers with BPD”)
- Mothers and their children must be in regular (weekly) contact or live together
- Sufficient German language competence
- Mothers with BPD must have completed or must be currently receiving therapy for their BPD symptoms.

Exclusion criteria:

- Acute child endangerment
- Acute suicidality
- Acute psychotic symptoms
- Acute alcohol or drug dependency
- Diagnosed intellectual disability.

Trainers delivering the intervention should:

- Be female
- Have either kids on their own
- Or be experienced in working with patients with BPD
- Be either licensed clinical psychotherapists or currently in training as clinical psychologists.

Having kids on their own helps trainers to understand the difficulties of participating mothers and to classify children’s behavior. As well, self-disclosure of the trainers regarding problems or doubts in child rearing facilitates mothers to open up.

Drop out criteria:

- Mothers who withdraw their participation after being included (after interview for eligibility)
- Mothers who cannot be reached within the defined time slots for post (week 12 + 2 months max.) and follow-up (month 9 + 2 months max.) assessments; if mothers miss out post assessment, they are still contacted for follow-up.

### Intervention

The manualized group-intervention “Parenting Skills for Mothers with Borderline Personality Disorder” [5, 6] is based on the concept of Dialectical Behavioral Therapy [37, 38]. DBT was developed to treat severe emotion dysregulation in BPD and focuses on the reduction of self-harming behavior, promotes mindfulness, and contains skills training to better cope with stress and high tension. BPD has proven to be effective in the treatment of BPD [2].

The group-intervention “Parenting Skills for Mothers with Borderline Personality Disorder” comprises 12 weekly 2 h-sessions covering the topics Psychoeducation, Mindfulness, Basic Needs of Children, Stress, Stress Management, Structure and Flexibility, Dealing with Conflicts, Dealing with Children’s and One’s Own Emotions, Physical Contact and Expression, Basic Assumptions about Parenting, and Self-Care. In the last session, all topics are summarized. Each session follows the same structure: mindfulness exercise, homework discussion, break, introduction into new topic using a practical exercise, homework for next session, take-home message. In-between sessions, participants are requested to practice newly gained techniques or knowledge in interaction with their children. Each session and the supporting material (i.e., information sheets and homework instructions) are described in detail in the manual.

#### Intervention—modifications

A Critical Incident Reporting System has been established. All serious adverse events (SAE) are documented. A consulting Data Safety Monitoring Committee (DSMC) is informed in cases of child or mother endangerment. The DSMC monitors all written reports of SAEs from the participating centers. Study exclusion only occurs in case of serious endangerment for the mother or child.

#### Intervention adherence

All group trainers receive a two-day training with the manual and role-play exercises by one of the manual’s authors. Frequent supervisions (every third session), video-based adherence checks, and adherence checklists after each session monitor adherence to the manual.

#### Intervention—concomitant care

As the intervention is conceptualized as an add-on parenting program, no restrictions are set regarding concomitant interventions as individual psychotherapy, skills training, or psychopharmacological medication.

### Outcomes

#### Primary outcome

Self-reported parenting is chosen as primary outcome as the intervention aims to reduce negative and enhance positive parenting. Additionally, child abuse potential is analyzed as primary outcome.

##### Primary outcome measures

Participants obtain a private link to complete primary outcome measures via web-based questionnaires at day 0 (before intervention), week 12 (directly after intervention), and month 9 (6 months after second assessment). Parenting strategies are assessed via the Brief Parenting Scale (German EFB-K; [39]) and the Alabama Parenting Questionnaire (German DEAPQ-EL-GS; [40]). Physical abuse potential is assessed using the Child Abuse Potential Inventory (CAPI; [41], German EBSK; [42]). Physical and psychological aggression towards the child is assessed via the Conflict Tactic Scale—Parent Child (CTS-PC; [43]) and the Child Neglect Index (CNI; [44]) filled out by the study staff and by the trainers in the intervention group after each session.

#### Secondary outcomes

In addition to direct effects on parenting and child abuse potential, changes in parental distress and perceived parental competencies are expected. Changes in borderline-specific psychopathologies (emotion regulation, mental distress, and borderline-specific thoughts and feelings) are also examined.

##### Secondary outcome measures

Participants rate their perceived parental competence and parental stress completing the Parenting Sense of Competence Scale (German EFB-K; 39) and the Parental Stress Index (German EBI; [45]).

Emotion regulation is assessed via the Difficulties in Emotion Regulation Scale (DERS; [46]), mental distress using the Brief Symptom Inventory (BSI; [47], German: [48]), and BPD-specific cognitions and emotions via the Questionnaire of Thoughts and Feelings (QTF; [49]).

All variables are assessed via web-based questionnaires on day 0 (before intervention), week 12 (after intervention), month 9 (6 months after second assessment).

#### Additional measures

To analyze potential control variables such as maternal trauma and conflicts in partnership, the Childhood Trauma Questionnaire (CTQ; [50], German version; [51]) at day 0 and the Conflict Tactic Scale for Partners (CTS-P; [52]) (day 0, week 12, and month 9) are applied. As an additional exploratory aspect, maternal mentalization is assessed using the Parental Reflective Functioning Questionnaire (PRFQ; [53]) (day 0, week 12, and month 9).

#### Additional outcomes in the intervention group only

After each session, trainers fill out the CNI (see above) and a form checking SAEs to check adverse events.

To assess unspecific intervention-related outcomes such as negative intervention effects and client satisfaction, a questionnaire assessing negative intervention outcomes (INEP; [54]) and the Client Satisfaction Questionnaire (CSQ-8; [55], German ZUF-8; [56]) are applied directly after the last session (week 12) in the intervention group only.

### Power analysis and sample size

Because the primary hypothesis concerns the investigation of the effect of the intervention in contrast to the waiting control group, power calculation is based solely on a repeated measure, within-between ANOVA. We strive for a statistical power of 99%; multiple-comparison problems are considered according to Cramer et al. [57]. A conservative estimate of *r* = 0.5 is used for the correlation between repeated measures of the same outcome to ensure enough power for outcomes with relatively low stability over time. Using the software G-Power, these specifications lead to a required sample size of 110 patients (2*55 per group intervention/waiting group) to detect an interaction when Cohen’s *f* for the interactions of 2*3 ANOVA is assumed to be moderate at 0.20. Based on our pilot groups, we assume that 10% of participants assigned to the intervention will not start intervention after assignment. Dropout rates from psychotherapy trials for BPD range between 22.3% and 28.2% [58]. In conclusion, we expect a dropout rate from baseline to follow-up assessment of 25%. Therefore, we assess *n* = 178 mothers with BPD for eligibility (*n* = 160 start the trial, *n* = 140 at post assessment, *n* = 120 at follow-up).

### Methods: assignment of interventions

Sequence generation: Mothers with BPS are randomly assigned after inclusion to either control or intervention group with a 1:1 allocation as per a computer-generated randomization schedule (RedCap software) stratified by site.

Concealment mechanism: Allocation concealment will be ensured, as study staff will not release the randomization results until the participant has passed the baseline assessments. If randomization is run before baseline assessments, study staff running the randomization differ from those conducting baseline assessments. Otherwise, randomization is run after baseline assessments, and participants are informed via telephone.

When sufficient participants are randomized to the intervention group, personal data (name and phone number) are passed on to the group trainers who contact the group participants. Group trainers do not have access to participants’ study data (diagnosis, questionnaires) or IDs. All intervention material is passed on to the study staff and allocated to the respective ID.

### Methods: data collection, management, and analysis

#### Preliminary statistical analyses

Descriptive statistics, correlations of variables, drop-out analyses, and randomization tests are analyzed first.

#### Statistical analyses of intervention effects

Analyses follow the intent-to-treat framework. In particular, we will use linear mixed effects models [59] with their advantages in the handling of missing and longitudinal data to analyze of our primary outcome measures. The respective statistical models will include fixed effects for time, intervention and the interaction of time and intervention as well as random effects for the participants.

Differences in the effectiveness of the intervention across three sites will be investigated. The factor site will be included in all statistical analyses concerning the primary and secondary outcome measures if there are substantive differences.

Additionally, the effect of concomitant interventions will be investigated.

### Methods: Data monitoring

Data monitoring is supervised by the Charité-BIH Clinical Study Center, which is independent of the study sponsor and has no competing interests. The trial will be stopped once the planned N is reached. Potential serious adverse events are monitored by the DSMC.

### Auditing

The coordinating center in Berlin provides organizational support and overview over all study related processes (e.g., enrollment, site status and other commitments). All processes of each subproject are determined in a standard operating procedure (SOP) and continuously reviewed by the study staff. Deviations from the SOP are documented (e.g., missing data, dropouts). Recurrent deviations are discussed in monthly meetings of all study staff members. Regular site monitoring visits (pre-study visits, initiation visits, and periodic monitoring visits) are conducted.

### Ethics and dissemination

#### Dissemination strategy

The research team will publish one primary outcome article, describing the effect of the group intervention on parenting and child abuse potential. Another publication will focus on the secondary gains achieved by the intervention, testing effects on the secondary outcomes named in the measurement section of this protocol. Additional analyses using variables assessed for the other subprojects will be published.

Exploratory analyses will be conducted to test for possible moderators (see above additional measures) of the intervention effect. If any variables make a meaningful contribution to explain the effects of the intervention on the primary or secondary outcomes, these analyses may result in further publications.

Results will be presented at national and international congresses and workshops.

#### Ethical issues

Ethical approval for this RCT was obtained by the ethics committee of the DGPs (No. RennebergBabette2019-07-29VADM). The study is conducted in accordance with the Helsinki Declaration. The research team members have made sure that the study respects the following ethical principles: all the personal data gathered are treated confidentially, written informed consent is collected, data are securely stored and the data will only be used for research purposes. Participation in this research study is voluntary. Participants are reminded of their rights to withdraw from the study without giving any reason. Data privacy is guaranteed: all the research data gathered during the project is identified using pseudonyms. Personal data is being kept under lock and is stored separately from research data.

Standard operating procedures for all study procedures have previously been defined. Communications and publications will not enable the identification of individual participants.

### Protocol amendments

Any modifications to the study protocol which may impact on the conduct of the study, potential benefit of the participants or may affect participant safety (e.g., changes of study objectives and study design, addressed population, sample sizes, SOPs) will require a formal amendment to the protocol. Amendments need to be agreed upon by the BMBF (Federal Ministry of Education and Research) and approved by the Ethics Committee (DGPs) prior to implementation.

Minor corrections and/or clarifications that have no effect on the way the study is to be conducted are protocolled.

### Patient involvement

The group intervention has been developed in a continuous dialog with mothers with BPD. Before setting the design of the study, we conducted two intervention groups with all interviews, questionnaires, and assessments to check for feasibility and participant’s feedback. Participant’s feedback was incorporated in the final design.

## Conclusion

Main aim of this RCT is to evaluate a newly developed, disorder-specific parenting intervention for mothers with BPD. Using a longitudinal design, we expect the intervention to have a positive effect on parenting and to decrease the risk of child abuse (primary outcomes). Additionally, we hypothesized the intervention to reduce parental stress, enhance parental competence, improve maternal emotion regulation, and reduce mental distress (secondary outcome).

We expect the intervention to enhance the opportunity for a healthy child development. At best, in the long run, an interruption of the transmission of violence and emotion regulation is achieved.

### Access to data—Intra study data sharing

A cleaned data set will be provided by the Charité-BIH Clinical Study Center. All Principal Investigators will be given access to the data sets. Project data sets will be transferred according to the standard operating procedures (SOPs) defined for the project. All data sets will be password protected and blinded of any identifying participant information.

### Ancillary and post-trial care

In case of ancillary or post-trial needs of participants, study staff provides information, contact possibilities, or access to consulting (e.g., family counseling) and psychotherapy treatment (e.g., in- or outpatient facilities).

Should this study provide evidence of the effectiveness of the training, efforts will be made to establish the intervention in in- and outpatient facilities for mothers with BPD.

## Data Availability

Parts of the final anonymized trial data set will be available.
